# Age and Sex Modulate SARS-CoV-2 Viral Load Kinetics: A Longitudinal Analysis of 1735 Subjects

**DOI:** 10.3390/jpm11090882

**Published:** 2021-09-02

**Authors:** Valerio Caputo, Andrea Termine, Carlo Fabrizio, Giulia Calvino, Laura Luzzi, Claudia Fusco, Arcangela Ingrascì, Cristina Peconi, Rebecca D’Alessio, Serena Mihali, Giulia Trastulli, Domenica Megalizzi, Raffaella Cascella, Angelo Rossini, Antonino Salvia, Claudia Strafella, Emiliano Giardina

**Affiliations:** 1 Genomic Medicine Laboratory UILDM, IRCCS Santa Lucia Foundation, 00179 Rome, Italy; v.caputo91@gmail.com (V.C.); andreatermine544@gmail.com (A.T.); carlo.fabrizio217@gmail.com (C.F.); giulia.calvino95@gmail.com (G.C.); lauraluzzi92@gmail.com (L.L.); fuscoclaudia19@gmail.com (C.F.); arcangelaingrasci@gmail.com (A.I.); cristinapeconi@gmail.com (C.P.); rebeccadal95@gmail.com (R.D.); mhlsrn95m42f611m@studenti.unical.it (S.M.); giulia.trastulli95@gmail.com (G.T.); domenica.megalizzi.96@gmail.com (D.M.); raffaella.cascella@gmail.com (R.C.); claudia.strafella@gmail.com (C.S.); 2 Department of Biomedicine and Prevention, Tor Vergata University, 00133 Rome, Italy; 3 Medical Services Direction, IRCCS Santa Lucia Foundation, 00179 Rome, Italy; a.rossini@hsantalucia.it (A.R.); a.salvia@hsantalucia.it (A.S.)

**Keywords:** COVID-19, SARS-CoV-2, viral load, subgenomic-RNA, age, sex, duration of infection, precision medicine

## Abstract

The COVID-19 pandemic caused by SARS-CoV-2 represents a public health emergency, which became even more challenging since the detection of highly transmissible variants and strategies against COVID-19 were indistinctly established. We characterized the temporal viral load kinetics in individuals infected by original and variant strains. Naso-oropharyngeal swabs from 33,000 individuals (admitted to the IRCCS Santa Lucia Foundation Drive-in, healthcare professionals and hospitalized patients who underwent routinary screening) from November 2020 to June 2021 were analyzed. Of them, 1735 subjects were selected and grouped according to the viral strain. Diagnostic analyses were performed by CE-IVD RT-PCR-based kits. The subgenomic-RNA component was assessed in 36 subjects using digital PCR. Infection duration, viral load decay speed, effects of age and sex were assessed and compared by extensive statistical analyses. Overall, infection duration and viral load differed between the groups (*p* < 0.05). Male sex was more present among both original and variant carriers affected with high viral load and showing fast decay speed, whereas original strain carriers with slow decay speed resulted in older (*p* < 0.05). Subgenomic-RNA was detected in the positive samples, including those with low viral load. This study provides a picture of the viral load kinetics, identifying individuals with similar patterns and showing differential effects of age and sex, thus providing potentially useful information for personalized management of infected subjects.

## 1. Introduction

The Coronavirus Disease 19 (COVID-19) pandemic caused by Severe Acute Respiratory Syndrome Coronavirus-2 (SARS-CoV-2) started a health crisis worldwide that still concerns public safety. In fact, more than 211,288,358 individuals have been infected by the SARS-CoV-2, causing the death of 4,422,666 people worldwide until August 2021 [1]. In particular, SARS-CoV-2 is able to cause a wide range of symptoms, including dry cough, fever, together with severe acute respiratory distress syndrome, acute kidney injury, secondary infections as well as cardiological and neurological manifestations [2,3,4]. To date, the strategies adopted in order to counteract viral spreading have been indifferently adopted for the entire population. However, the heterogeneity of symptoms and of their severity points out the need of investigating factors underlying the differential response to the infection and to the disease. The identification of groups of individuals with specific features would enable the set-up of personalized approaches addressed to improve the management, effectiveness and safety of treatments. SARS-CoV-2 is characterized by a positive-sense single-stranded RNA genome of about 30 kb that replicates employing the transcription of negative-sense RNA intermediates, providing templates for the synthesis of novel genomes as well as various subgenomic RNAs (sg-RNAs) [5]. Similar to other coronaviruses, this replicative mechanism is thought to allow these viruses to rapidly evolve by introducing genetic mutations, some of which can enhance their transmissibility, can complicate viral identification through routine diagnostic protocols or can affect the effectiveness of treatment and vaccination. To date, several viral strains have been reported. In Italy, the predominant clades from September 2020 to January 2021 were represented by GISAID clade G (lineage B.1, Nextstrain clade 20A) and related clades GR (lineage B.1.1.1, Nextstrain clade 20B and lineage C, Nextstrain clade 20D) and GV (lineage B.1.177, Nextstrain clade 20E, EU1) [6,7,8]. Moreover, different Variants Of Concerns (VOCs) occurred, with the alpha variant (αV) (lineage B.1.1.7, GISAID clade GRY, Nextstrain clade 20I/501.Y.V1) and the beta variant (lineage B.1.351, GISAID clade GH, Nextstrain clade 20H/501.Y.V2) being the most relevant for public safety [9]. In particular, the αV dramatically raised in the first part of 2021, whereas the delta variant (δV) (lineage B.1.617.2, GISAID clade G/478K.V1, Nextstrain clade 20A/S.478K) recently showed a dramatic increase, raising the need of maintaining the measures to counteract the spreading of infection. In this context, studies aimed at characterizing the clinical samples from infected individuals in terms of viral load kinetics during the time of infection are necessary to verify the presence of differential interaction of individuals with the viral strains, which may contribute to their spreading and thereby being used for developing patient-based counteracting measures. Thus, we performed a single-center longitudinal study including a selection of positive naso-oropharyngeal samples analyzed during the second and third wave of the COVID-19 pandemic at the Genomic Medicine UILDM laboratory of Santa Lucia Foundation Scientific Institute for Research, Hospitalization and Healthcare (IRCCS) in Rome (Italy). In particular, the study included 33,000 Italian individuals admitted to the IRCCS Santa Lucia Foundation Drive-in, healthcare professionals and hospitalized patients who underwent routinary screening, sampled from November 2020 to June 2021. During this time, the diffusion of the G, GR and GV clades (referred to as Original Strains, OSs, from this point on) and the occurrence and rapid spreading of VOCs, offered the chance to identify differences in viral kinetics among the affected patients. Indeed, the identification of a differential response to the virus may contribute to personalized management and treatment of infected patients.

## 2. Materials and Methods

### 2.1. Selection of the Cohort

Naso-oropharyngeal swabs from 33,000 individuals were collected at the IRCCS Santa Lucia Foundation from 17 November 2020 to 31 June 2021 and were evaluated for the study. Of them, 1735 subjects were selected for downstream analysis based on the following criteria: (i) at least 3 consecutive positive tests; (ii) the time interval between each test had to be equal to 7 ± 2 days. The participants provided written informed consent. The study was performed according to the Declaration of Helsinki.

### 2.2. Molecular Diagnosis of SARS-CoV-2

Naso-oropharyngeal samples were obtained using cotton swabs in Universal Transport Medium (UTM) (Copan Diagnostics, Brescia, Italy). Molecular diagnosis of SARS-CoV-2 infection was performed by purifying viral RNA from 300 μL of UTM through automated extraction by Magpure virus DNA/RNA purification kit (Hangzhou Bigfish Bio-tech Co. Ltd. Hangzhou, China) on Nuetraction 32 Nucleic Acid Purification System (Hangzhou Bigfish Bio-tech Co. Ltd.) according to manufacturer’s instructions. The extracted RNAs were subjected to one-step Real Time-PCR (RT-PCR) by means of TaqPath COVID-19 RT PCR CE IVD kit (ThermoFisher Scientific, Waltham, MA, USA) using QuantStudio 5 RT-PCR system according to manufacturer’s instructions [10]. Resulting data were analyzed and interpreted using the QuantStudio DA2 and COVID-19 Interpretive Softwares (ThermoFisher Scientific).

### 2.3. Identification of SARS-CoV-2 VOCs

Samples tested by the RT-PCR test, which reported the S gene-target failure and the presence of only N and Orf1ab targets were considered as suggestive of infection by the αV, as described in the literature [11]. The identification was then confirmed by means of RT-PCR using the COVID-19 Variant Catcher CE IVD kit (Clonit, Siziano, Italy). In particular, assays specific for three mutations within the S gene (HV 69-70 deletion, E484K and N501Y) are employed in order to differentiate the αV from the OSs or beta/gamma variants. To this purpose, 5 µL from the extracted RNAs were tested on QuantStudio 5 RT-PCR system (ThermoFisher Scientific) according to manufacturer’s instructions. The presence of δV was assessed by whole genome sequencing performed by INMI Lazzaro Spallanzani (Rome, Italy) during the molecular surveillance program.

### 2.4. Assessment of Viral Subgenomic-mRNA

The presence of sg-RNA was assessed by specific retrotranscription (see [12]) and digital PCR (dPCR) in 122 longitudinal RNA samples from a sub-cohort of 36 individuals characterized by age, sex distribution and basal viral load compared to those of the whole cohort. The resulting cDNAs were then tested by means of chip-based dPCR using TaqMan assays specific for N and E genes on QuantStudio 3D Digital PCR System (Life Technologies, Carlsbad, CA, USA). In particular, 6 µL of each cDNA was combined with QuantStudio™ 3D Digital PCR Master Mix (Life Technologies) and the TaqMan assay specific for N or E gene and, then, loaded on QuantStudio™ 3D Digital PCR Chips according to manufacturer’s instructions. Each sample was tested in duplicate on two different chips. Loaded samples were then amplified on ProFlex PCR system (Life Technologies) and the end-point fluorescent data were analyzed by QuantStudio™ 3D Digital PCR Instrument (Life Technologies) and the QuantStudio™ 3D AnalysisSuite™ (Life Technologies).

### 2.5. Statistical Analyses

In order to uniform the starting point in the longitudinal analysis, the 2nd positive test for each subject was fixed as “T0”. Viral load was computed in log_10_ (copies/mL) at each time point for each individual. Viral Load Decay Speed (VLDS) was calculated as the viral load slope between T0 and T1 (i.e., extracting the angular coefficient of the regression line built for viral load over time at subject level). The duration of the infection was defined as the time interval in days between the first negative test after two positive tests. Considering that these diagnostic tests took place in the second part of 2020 and in the first five months of 2021, we split this cohort according to the SARS-CoV-2 strain in two groups, indicative of the infection by OSs and αV. The differences between groups in terms of duration of the infection, age and VLDS were assessed by Welch *t*-test [13], whereas sex differences in the group composition were tested by Chi-square [14,15]. A Generalized Linear Mixed Model (GLMM) was used to assess differences in viral load over time between the groups [16]. Subjects were stratified into three classes (Fast, Medium, Slow) based on their decay speed using quantile split (Fast ≤ −2.40 (33%); −2.40 < Medium < −1.36; Slow ≥ −1.36 (67%) [17]. After this comparison, longitudinal studies were performed to characterize αV and OSs groups. Considering that VLDS may be affected by viral load at T0, we decided to test this potential association for both groups. Viral load at T0 was used to stratify the subjects into three classes (Low, Medium, High) using quantile split (Low ≤ 2.4 (33%); 2.4 < Medium < 3.45; High ≥ 3.45 (67%) [17]. Differences in VLDS were tested between basal load classes using Welch ANOVA [18] with pairwise Games–Howell tests for post-hocs [19] and Holm-correction [20]. The influence of the VLDS on the duration of infection was tested using a GLMM with decay speed classes as a fixed effect and the viral load class at T0 as a random effect. Post hoc were performed using Tukey-HSD [21] with Satterthwaite correction [22]. Similarly, age effects on VLDS were tested using a GLMM with baseline viral load as a random effect. Sex differences in VLDS classes were assessed for each viral load class using Fisher’s Tests [14]. In order to compute achieved statistical power and effect size on duration of infection and age, a two-tailed post-hoc power analysis for *t*-tests was performed and Cohen’s d was obtained. Additionally, effect sizes for sex differences were computed by means of Cohen’s w [23].

VLDS of the δV patient was assessed in the context of a reference group composed of 112 individuals matched for age (12 < Age < 22) and sex. Grubbs test was used to test whether the δV patient represented an outlier in terms of VLDS [24].

## 3. Results

### 3.1. Comparison of OSs and αV Carrier Individuals

**Figure 1:**
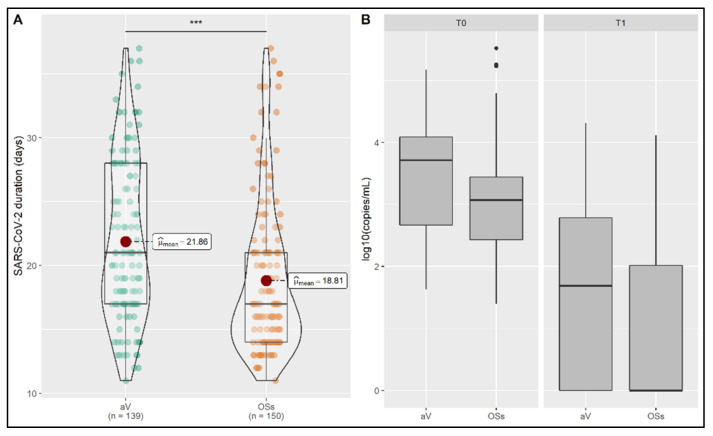
Comparison of OSs and αV carrier individuals. (**A**) The duration of infection significantly differed between OSs and αV carriers. The average day of resolution of infection is reported for each group. (**B**) Viral load and VLDS are shown between groups. Carriers of αV were found with a higher viral load (*p* < 0.03); *** *p* < 0.001.

The selected 1735 subjects were subdivided into two groups according to the infection by the OSs (*n* = 1581) or αV (*n* = 154) of SARS-CoV-2. We first compared them in terms of viral load, VLDS, gender and age. In particular, individuals infected by αV were found to resolve the infection in averaged 21.86 (sd = 6.22) days, whereas those infected by OSs were characterized by a duration of infection of averaged 18.81 (sd = 5.82) days (Figure 1A). The effect size (indicated by Cohen’s d) was d = 0.51, with achieved statistical power of 0.99. A GLMM was fitted to assess the effect of time and viral strain (OSs/αV) on viral load. In this way, individuals infected by αV were found with a significantly higher viral load (*p* < 0.03) (Figure 1B). VLDS did not significantly differ between the groups. Age and gender were equally distributed in both groups. In particular, the average age was 39.55 (sd = 18.68) and 40.30 (sd = 19.26) for OSs and αV groups respectively, whereas the male gender was equal to 49% in the OSs carriers and 51% in the individuals carrying the variant strain. VLDS was not significantly different.

### 3.2. Analysis of OSs Carriers Group

**Figure 2:**
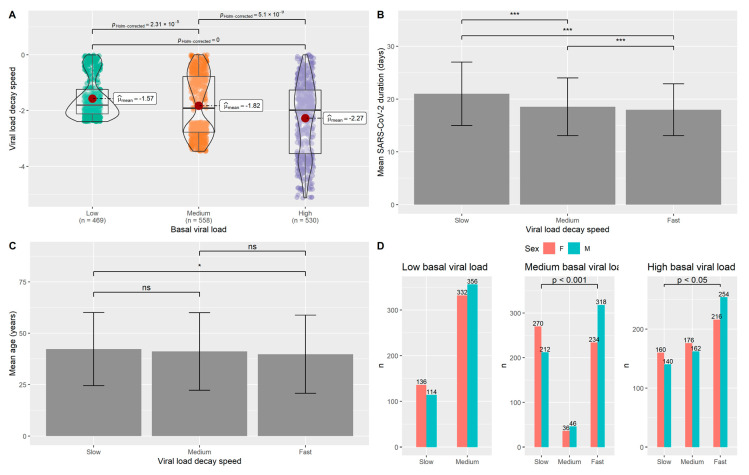
Analysis of the OSs carriers group. (**A**) VLDS showed to be dependent on the basal viral load, proving the need of stratifying according to viral load inside the group. (**B**) The duration of infection significantly differs among individuals with different VLDS. (**C**) Age was found to be significantly different between individuals with low and high viral loads. (**D**) Sex distribution was different among individuals with different ranges of viral load. Male sex was found to be significantly present in the group of individuals with high and medium viral loads characterized by fast VLDS. *n*= number of subjects; * *p* < 0.05; *** *p* < 0.001.

Concerning the group composed of individuals carrying the OSs, the VLDS showed to depend on basal viral load and be determinant for the duration of infection, as demonstrated by the significant differences in all the comparisons between the classes (Figure 2A,B). The calculated effect sizes were d = 0.43; d = 0.55 and d = 0.10 with a statistical power of 0.99, 1.00 and 0.39 for the comparison slow VLDS vs. medium VLDS, slow VLDS vs. fast VLDS and medium VLDS vs. fast VLDS, respectively (Supplementary Materials: Table S1). Individuals in the slow-speed group showed to be older than those in the fast-speed group (Figure 2C). The Cohen’s d calculated for this comparison was equal to 0.13 with an achieved statistical power of 0.61. Moreover, among high and medium viral load groups, there was a higher frequency of male subjects in the fast VLDS group when compared to the slow VLDS group (*p* < 0.001 and *p* < 0.05, respectively) (Figure 2D). Cohen’s ws were calculated, obtaining w = 0.28 (statistical power= 1.00) for the comparison between slow VLDS and fast VLDS individuals with medium viral load, and w = 0.15 (statistical power = 0.90) for the same comparison between individuals with high viral load (Supplementary Materials: Table S2).

### 3.3. Analysis of the αV Carriers Group

**Figure 3:**
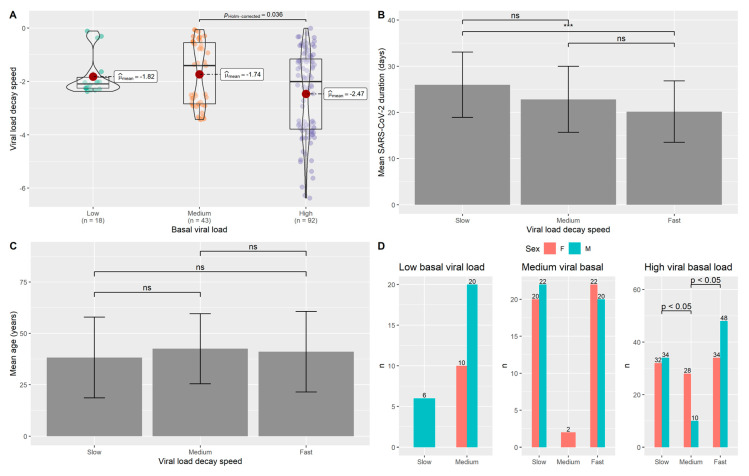
Analysis of the αV carriers group. (**A**) VLDS showed to be dependent on the basal viral load, proving the need of stratifying according to viral load inside the group. (**B**) The duration of infection significantly differs between individuals with slow and fast VLDS. (**C**) Age did not show a significant difference with respect to individuals with different VLDS. (**D**) Sex distribution was different among individuals with a high viral load. Male sex was found to be significantly different between the individuals characterized by fast and medium as well as slow and medium VLDS. *n*= number of subjects; *** *p* < 0.001; *ns*: not significant.

Similar to the OSs carriers, the VLDS related to the αV carriers was found as dependent on the basal viral load and determinant for the duration of infection (Figure 3A,B). A significant difference was found between medium and high basal viral load classes. The duration of infection was dependent on the basal viral load, with a significant difference between individuals with slow and fast decay of viral load (Figure 3B). Cohen’s d = 0.84 with a statistical power of 0.99 was obtained for this comparison. Age did not reach statistical significance as a contributor to different VLDS (Figure 3C). The VLDS groups were differentiated by sex composition. In fact, sex reached a statistical significance in the group of individuals with high viral load, in which male sex was prevalent among individuals with fast and slow VLDS compared to medium VLDS (Figure 3D). Cohen’s w values were computed and a w = 0.50 (statistical power= 0.98) and a w = 0.73 (statistical power= 0.99) were obtained for the comparisons between slow VLDS and medium VLDS as well as medium VLDS vs. fast VLDS individuals with high viral load, respectively (Supplementary Materials: Table S2).

### 3.4. Analysis of δV Carrier

An infected subject was found to be a carrier of the emerging δV in June 2021. The viral load, VLDS and the time of infection duration were compared with those of age and sex-matched carriers of the OSs and αV. According to these analyses, the δV carrier did not show significant differences.

### 3.5. Detection of sg-RNA 

The presence of sg-RNA related to N and E genes was assessed in clinical samples from a sub-cohort of 36 subjects. In particular, N was detected with a concentration ranging from 1.88 to 7.39 log_10_(copies/mL) and E from 1.87 to 7.30 log_10_(copies/mL). Both sg-RNAs were detected until an average day of 28.6 (sd = 11 days). 

**Figure 4:**
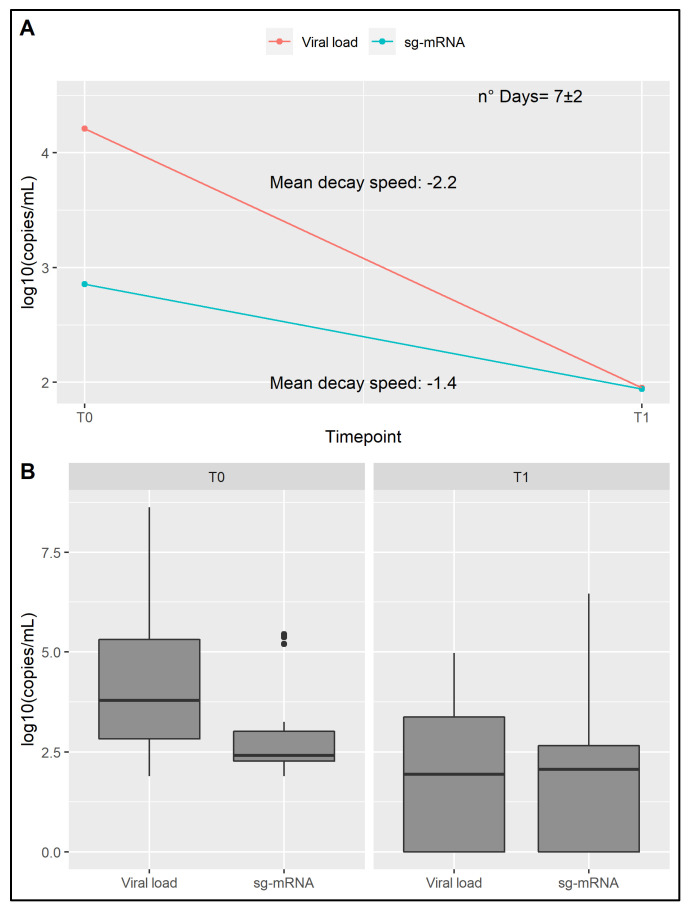
Assessment of sg-RNA component. (**A**) Representation of the sg-RNA concentration and corresponding viral load decay over time. (**B**) sg-RNA concentration and corresponding viral load reported for the deemed timepoints. Both sg-RNA concentration and viral load are reported in log_10_ copies/mL.

Considering the standardization of timepoints as previously described, the range of sg-RNA, intended as the average of N and E log_10_(copies/mL), was from 1.87 to 6.47 (Figure 4A,B).

## 4. Discussion

During the routinely diagnostic activity, our laboratory was able to perform more than 60,000 diagnostic tests aimed at detecting SARS-CoV-2 infection. Since the end of 2020, the occurrence and diffusion of viral variants characterized by increasing transmissibility were observed, including the αV, which rapidly induced several concerns for public health [25]. Precautionary strategies against the diffusion of SARS-CoV-2 were adopted, without considering specific and individual responses to the infection. In this context, the present study aimed at providing an overview of infection, which could help in setting strategies for personalized management of patients infected by SARS-CoV-2. The pattern of infection was assessed in terms of viral load and VLDS, grouping the subjects according to the levels of viral load (low, medium and high), VLDS (slow, medium and fast) and duration of infection. Extensive statistical analyses were performed in order to compare those individuals infected by the OSs, which were predominant in the first months of this survey (September 2020–January 2021) and those affected by the αV which reached 80% of total infections in Italy in the second part of the survey period (February 2021–June 2021) [26]. Since WGS data were not available for the OSs group, this cohort may have included different SARS-CoV-2 clades. Therefore, OSs individuals were likely to be carriers of G clade and its descendents GR and GV, considering their predominance in Italy during the second part of 2020. In particular, since we included samples from November 2020, it is likely that these samples mostly carried the GV clade, which rapidly spread throughout the country and reached a frequency of 96% in December 2020 [6,8]. OSs and αV groups were found to be almost similar in terms of age and gender composition. Nevertheless, we were able to observe interesting differences between these cohorts. As a matter of fact, higher viral load and long-lasting infection were found for those carrying the αV, as also shown by the achieved medium effect size in the present study and in literature [27]. However, the viral load decay speed was not significantly different between the cohorts, suggesting that the speed may be dependent on the basal viral load independently of the SARS-CoV-2 strain (Figure 2A,B). In line with this result, other studies evaluating the correlation between viral load and its decay over time found that higher amounts of virus detected in the naso-oropharyngeal swabs were correlated to an increased viral persistence [27,28]. Moreover, the duration of the infection significantly differed between OSs carriers with different VLDS and these data were reinforced by the medium effect size obtained by comparing slow VLDS vs. medium/fast VLDS individuals (Supplementary Materials: Table S1).

Successively, we aimed at classifying individuals with specific times of infection resolution, considering the VLDS. To this purpose, subjects were sub-grouped according to the basal viral load in order to avoid bias linked to lower or higher viral loads. In the group of OSs carriers, age appeared as a discriminating factor, with older individuals characterized by a slower VLDS, suggesting that physiological aging may represent a contributing factor for the different timing of the duration of infection. Interestingly, this data points out the occurrence of differences among younger and older people in our cohort, which should be taken into account for effective management of the outbreak, also by personalizing the management of patients according to the age-related risk of infection, transmissibility and progression to severe disease. In fact, age-related physiological impairment was shown to affect both innate and adaptive immune responses, which may lead to higher susceptibility to infections. In particular, immunosenescence was found to be related to the susceptibility to infection with SARS-CoV-2 and to the severity of disease [29]. Therefore, the age-related functional decline of immune-inflammatory response may also exert a role in the viral persistence inside the organism and contribute to the differential response to vaccination. Of note, the OSs group is composed of relatively young people (characterized by a medium age of 40 years) and thus the small difference detected among individuals with slower and faster VLDS (2.57 years) and the resulting negligible effect size (Figure 2C) may be due to this feature. Indeed, during the study period, younger people were more likely to move and travel and, thus, they were more exposed to the infection.

Moreover, it is definitely possible that VLDS may also depend on other host factors. On this subject, significantly different sex distributions were found among individuals with the same VLDS, with small and medium effect sizes obtained considering individuals with medium and high viral load, respectively (Supplementary Materials: Table S2). Notably, a gender-specific difference in the susceptibility to many infective viruses is well known [30], although a similar incidence for males and females was reported regarding the infection by SARS-CoV-2. However, an effect of male sex in the progression to severe COVID-19 was postulated [31].

This divergence may be contributed by genetic processes such as the X-chromosome inactivation and escaping, molecular mechanisms related to sex hormones, as well as habits and lifestyle. In fact, gender-specific patterns of gene expression due to hormone regulation and differential regulation of immune factors might contribute also to the host responses against the virus and the timing of the duration of the infection. In particular, estrogens were found to enhance the production of regulatory T-lymphocytes. Accordingly, cell-mediated immunity responses can be higher in females compared to males. On this subject, females were reported to have a greater amount of immune cells (such as antigen-presenting cells, APCs, and monocytes) [32]. Moreover, some genes playing important roles in immune signaling are located within the X-chromosome, including *IGSF1*, *IL9R*, *IRAK1*, *TLR7* and *TLR8* and different miRNAs regulating immune response, such as miR-223, miR-221/222 cluster, miR-98, miR-106a, miR-424, miR-18b and miR-548am-5p [33,34]. Intriguingly, *TLR7* and different miRNAs, including miR-188, miR-421, miR-503, Let-7f2, and miR-98, were reported to escape from X-inactivation, a mechanism that could explain their higher expression in female subjects [33]. In fact, these miRNAs were found overexpressed in CD4 T-cells from females affected by Systemic Lupus Erythematosus, and this mechanism was proposed as a contributing factor to the differential predisposition to this autoimmune condition [35]. Therefore, further studies could be performed in order to characterize the effect of sexual dimorphism in the inner responses against SARS-CoV-2 and the influence on the infection duration.

Moreover, host factors other than age and sex may also account for interindividual differences in terms of decay of viral load and for differential human interaction with viral variants. On this subject, the individual (epi)genetic make-up was investigated looking for specific variants and genes able to contribute to the susceptibility and progression to severe forms of COVID-19 [36]. In particular, an interesting role of immune genes (such as the *HLA* make-up) in the differential activation of host immune responses by SARS-CoV-2 variants was investigated, pointing out the importance of specific virus-host molecular interactions [37]. Therefore, studies at (epi)genome-wide level will certainly help to characterize groups of individuals with the same pattern of viral kinetics, providing useful insights for a more tailored and effective management of infected patients.

Concerning the αV group, significant differences with a large effect size in the duration of the infection were found between individuals with fast and slow decay speed, similar to the OSs group. In contrast to OSs carriers, age did not appear to influence the decay speed in the variant carriers group, although the lack of significance may be due to the lower sample size available for this study and, therefore, need to be further investigated.

Interestingly, sex distribution resulted to be different in the αV group, with male sex being more prevalent among the individuals with high viral load characterized by fast VLDS, as shown by the large effect sizes obtained (Supplementary Materials: Table S2). This data supports the potential role of sexual dimorphism in influencing the decay speed and probably the response to infection.

Considering the importance of understanding the risk of transmission, we performed a preliminary analysis in order to assess the quantity of sg-RNA in naso-oropharyngeal swabs. Overall, we detect sg-RNA in swabs characterized by low viral load, generally related to a later stage of infection. This data raises questions concerning the potential role of sg-RNA as a biomarker of an active infection. As a matter of fact, the production of sg-RNAs from the viral genome represents a fundamental step for viral replication, protein translation and assembling of infectious virions [38]. Interestingly, it was proposed as a hallmark of active replication, although this is still a controversial matter [39,40]. In fact, certain studies have reported the detectability of sg-RNA at 5 days post-infection in throat swabs [39]; whereas others, consistent with our results, detected sg-RNA in swabs until the 17th–22th day after the onset of infection, thus suggesting that this component may not be informative of the infection capability [40,41]. These results may depend on the features of the enrolled infected individuals at the time of molecular analysis (for instance, severity of symptoms, duration and stages of the disease) and on the employed technology. On this subject, we decided to use a very sensitive method such as dPCR, which allowed the detectability of low copies/mL of sg-RNA also in samples from late stages of infection. Further research is needed to clarify if sg-RNA or its specific amount levels may be suggestive of the presence of a viable virus and thus may be related to the higher/lower risk of viral transmission. This could unveil interesting insights concerning the characterization of different viral variants. In the present study, it was not possible to perform a comparison among OSs and variant carriers because of the sample size of the sub-group. In fact, a power analysis revealed the need for at least 944 subjects in order to provide a reliable comparison. Therefore, large-scale characterization of sg-RNA levels is required to evaluate potential specific features of the transmission capability related to SARS-CoV-2 variants, which is important regarding the ongoing emergence and rapid diffusion of highly transmissible viral variants.

Furthermore, an individual infected by the emergent δV was detected in June 2021. The assessment of potential differences in the pattern of viral load kinetics, by comparison of the patient with other sex- and age-matched αV and OSs carriers did not reveal features. However, this explorative analysis needs to be verified by sampling more *δ*V carriers.

We were not able to analyze the effect of vaccination on viral load kinetics, which indeed started to be provided to the Italian population only in the second part of the study. However, we recently assessed the effect of vaccination on the viral load decay in a vaccinated subject who resulted positive to SARS-CoV-2 αV. This patient underwent a more rapid resolution of the infection when compared to non-vaccinated αV and OSs carriers [12], suggesting that vaccination may exert an effect on the decay of viral load and, thus, on the transmission and time of infection resolution.

In conclusion, this study aimed at verifying the presence of differential interaction with the virus among patients positive to SARS-CoV-2 admitted at our center in a year of diagnostic testing. During this period, we observed not only the diffusion of the OSs but also the emergence and diffusion of different viral VOCs, including the αV and more recently the δV. Thanks to the extensive analyses, we were able to assess the VLDS and to analyze it in terms of variant type, age and sex. Thus, we identified groups of individuals with a similar pattern of viral kinetics and reported a potential influence of age and sex in the contribution to the detected differences. Moreover, we performed a preliminary analysis of the sg-RNA component, which was detectable even in samples corresponding to the later stages of infection. We were also able to characterize a single case of δV.

Of note, this study provided an initial characterization and stratification of infected individuals, which should be intended as a first step towards the development of more tailored management of infection by means of precision medicine approaches. To enable the application of such protocols, this characterization should be extended in the future by considering other instrumental data, such as the antibody titer, the assessment of immune-inflammatory status, the presence and severity of symptoms, epidemiological information (density of population in the community of interest, presence of close contacts, disease prevalence) and wide molecular and genomic studies. Such studies will also be useful to better assess the effectiveness of vaccination, revealing specific patterns and features that may explain the differential response to vaccines and duration of protection as well as the risk of viral infection and transmission related to the vaccinated individuals. Indeed, the availability of such data will be crucial to address more efficient strategies and improve the management of immunization campaigns.

## Data Availability

The data described in the present study are included within the manuscript and the Supplementary Materials.

## Supplementary Materials

The following are available online at https://www.mdpi.com/article/10.3390/jpm11090882/s1, Table S1: *Post Hoc* power analyses for the duration of the infection in the OSs group stratified by VLDS. Table S2: *Post Hoc* power analyses for sex differences.
