# Supplementary material for: Age and Sex Modulate SARS-CoV-2 Viral Load Kinetics: A Longitudinal Analysis of 1735 Subjects
Source: J Pers Med. 2021 Sep 2;11(9):882. doi: 10.3390/jpm11090882 (PMC8470027; doi:10.3390/jpm11090882)
Supplement: Supplementary file 1 [file jpm-11-00882-s001.zip › jpm-1352166-supplementary.pdf]

Supplementary Tables

Supplementary Table S1. *Post Hoc* power analyses for the duration of the infection in the OSs group stratified by VLDS.

Comparison	Effect size (Cohen's d)	Statistical power
Slow *vs* Medium	0.43	0.99
Slow *vs* Fast	0.55	1.00
Medium *vs* Fast	0.10	0.39

Groups sizes (Slow = 481, Medium = 530, Fast =490) after outlier removal, Group Means (Slow = 20.99, Medium = 18.54, Fast= 17.9), Groups SD (Slow = 6, Medium = 5.45, Fast = 4.91).

Supplementary Table S2. *Post Hoc* power analyses for sex differences

Group	Basal Viral load	Comparison	Effect size (Cohen's w)	Statistical power
OSs	Medium	Slow *vs* Fast	0.28	1.00
	High	Slow *vs* Fast	0.15	0.90
αV	High	Medium *vs* Slow	0.50	0.98
	High	Medium *vs* Fast	0.73	0.99

All the power analyses were performed testing the independence assumption. The female and male proportions in the samples were used in the contingency table.
